# Bioactive hierarchical silk fibers created by bioinspired self-assembly

**DOI:** 10.1038/s41467-021-22673-4

**Published:** 2021-04-22

**Authors:** Linpeng Fan, Jing-Liang Li, Zengxiao Cai, Xungai Wang

**Affiliations:** Institute for Frontier Materials, Deakin University, Geelong, VIC Australia

**Keywords:** Biophysics, Materials science, Biomaterials

## Abstract

Artificial recapitulation of the hierarchy of natural protein fibers is crucial to providing strategies for developing advanced fibrous materials. However, it is challenging due to the complexity of the natural environment. Inspired by the liquid crystalline spinning of spiders, we report the development of natural silk-like hierarchical fibers, with bundles of nanofibrils aligned in their long-axis direction, by self-assembly of crystallized silk fibroin (SF) droplets. The formation of self-assembled SF fibers is a process of coalesced droplets sprouting to form a branched fibrous network, which is similar to the development of capillaries in our body. The as-assembled hierarchical SF fibers are highly bioactive and can significantly enhance the spreading and growth of human umbilical vein endothelial cells compared to the natural SF fibers. This work could help to understand the natural silk spinning process of spiders and provides a strategy for design and development of advanced fibrous biomaterials for various applications.

## Abstract (web summary)

The creation of silk fibres using bioinspired approaches is of interest for biomaterials development. Here, the authors report on the creation of mimetic hierarchical silk fibres by the rotational self-assembly of silk fibroin droplets and demonstrate the creation of bioactive silk materials.

## Introduction

**Fig. 1:**
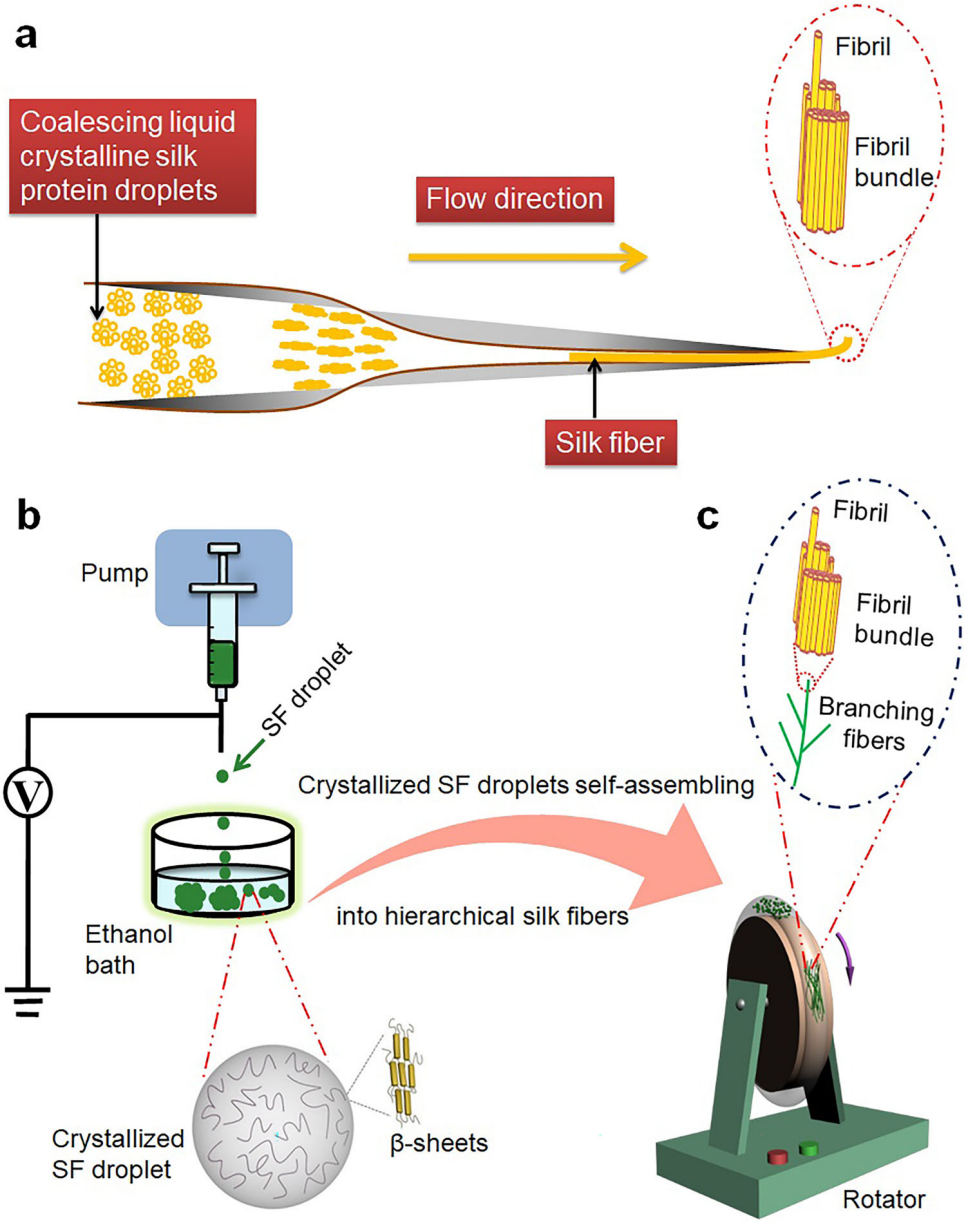
Schematic illustration of crystallized droplets of silk protein molecules self-assembling to hierarchical suprafibrillar silk fibers. **a** Schematic mechanism illustration of the liquid crystalline model for formation of in vivo silk fibers, which was drawn based on the references^10,11,15^. **b** Formation of crystallized droplets from silk fibroin (SF) molecules. **c** Crystallized SF droplets self-assembling to hierarchical suprafibrillar silk fibers.

Natural protein fibers such as collagen fibers, hairs, and silks play an essential role in the different phases of life cycle by supporting, stabilizing, and protecting cells, tissues, and organisms^1^. Interestingly, this type of protein fibers have a common structure with bundles of smaller fibrils in their long axis^2–6^. In terms of silks, one single silk fiber is a bundle of thinner fibrils aligned along its long axis and the width of the fibrils ranges from nanometers to sub-micrometers (Fig. 1a)^6–8^. The neighboring fibrils interact with each other via relatively weak interactions so that they can be isolated from silk fibers through simple treatment^9^, which is similar to that of the supramolecular system and thus we call it suprafibrillar structure. On a nanometer scale, one silk fibril is a network of β-sheet crystals interlinked by amorphous chains^6^. The single fibrils can be considered as domains of the supramolecular structure/network. In vitro recapitulation of the hierarchical suprafibrillar structure of natural silk fibers is crucial to understanding the in vivo assembly process and providing insight into the design and development of advanced artificial biomaterials.

For the formation mechanism of natural silk fibers, two classic models, i.e., the micelle model and the liquid crystalline model, have been proposed^10,11^. In the micelle model, silk protein molecules initially form micelles, which self-assemble into big globules with loss of water and are then spun into silk fibers^10^. The micelle model has been explained by the water–protein, protein–protein, and protein–polymer (PEO) interactions, and the wet-spinning process of aqueous silk fibroin (SF) solution^10^. Subsequently, different biomimetic spinning systems based on this model have been developed to fabricate various kinds of silk fibers in vitro^12,13^. Different from the micelle model, the liquid crystalline model proposes that the silk protein matrix in the silk gland contains numerous small spherical droplets with liquid crystalline property, which grow by coalescence and are subsequently spun into silk fibers (Fig. 1a)^11^. The classic liquid crystalline model is based on the presence of liquid crystalline droplets, which is proposed by analyzing the structure of spiders’ gland tissue as well as the structural, morphological, and compositional transformation of silk proteins in different segments of the gland^11^. Unfortunately, it is hard to recapitulate the hierarchy of natural silk fibers in vitro on the basis of this model due to the complexity of in vivo environment. In a recent study, bundled silk fibers were wet-spun from nematic solutions of silk microfibrils that were isolated from natural silk fibers^14^. However, the study used the microfibrils as building blocks and thus cannot give enough insight into the formation of hierarchical silk fibers from silk protein molecules or their crystalline droplets. Currently, the assembly of liquid crystalline droplets of silk protein molecules into hierarchical suprafibrillar silk fibers remains largely unexplored.

Here, we aim to understand the self-assembly process of crystallized droplets of silk protein molecules to form hierarchical suprafibrillar silk fibers and provide strategies for development of advanced fibrous biomaterials in vitro (Fig. 1). In this work, the crystallized droplets are fabricated by electrospraying droplets of aqueous SF solution into an ethanol bath in which the amorphous structure of SF with dominant random coils is induced to a β-sheet-rich crystalline structure (Fig. 1b). Subsequently, the crystallized droplets are induced to self-assemble by a circular rotary system (Fig. 1c). Differing from the spinning system in which the fibers are extruded instantly via the spinneret, here, the circular assembly system focuses on the self-assembly of crystallized SF droplets to form hierarchical silk fibers. Compared to the spinning process, one great advantage of the circular assembly system is that it allows us to easily monitor the entire assembly process of silk fibers by investigating the resultant structures at different assembly stages (such as different time points of the self-assembly process). This is also very important for obtaining resultant materials with tailored morphologies and structures. Additionally, with this self-assembly system, hierarchical silk fibers with width and thickness down to nanometer scale can be produced at a very low concentration of SF solution (0.2% (wt/v) in this work), which is very challenging to achieve using the existing technologies.

## Results and discussion

### Fabrication of crystallized SF droplets

**Fig. 2:**
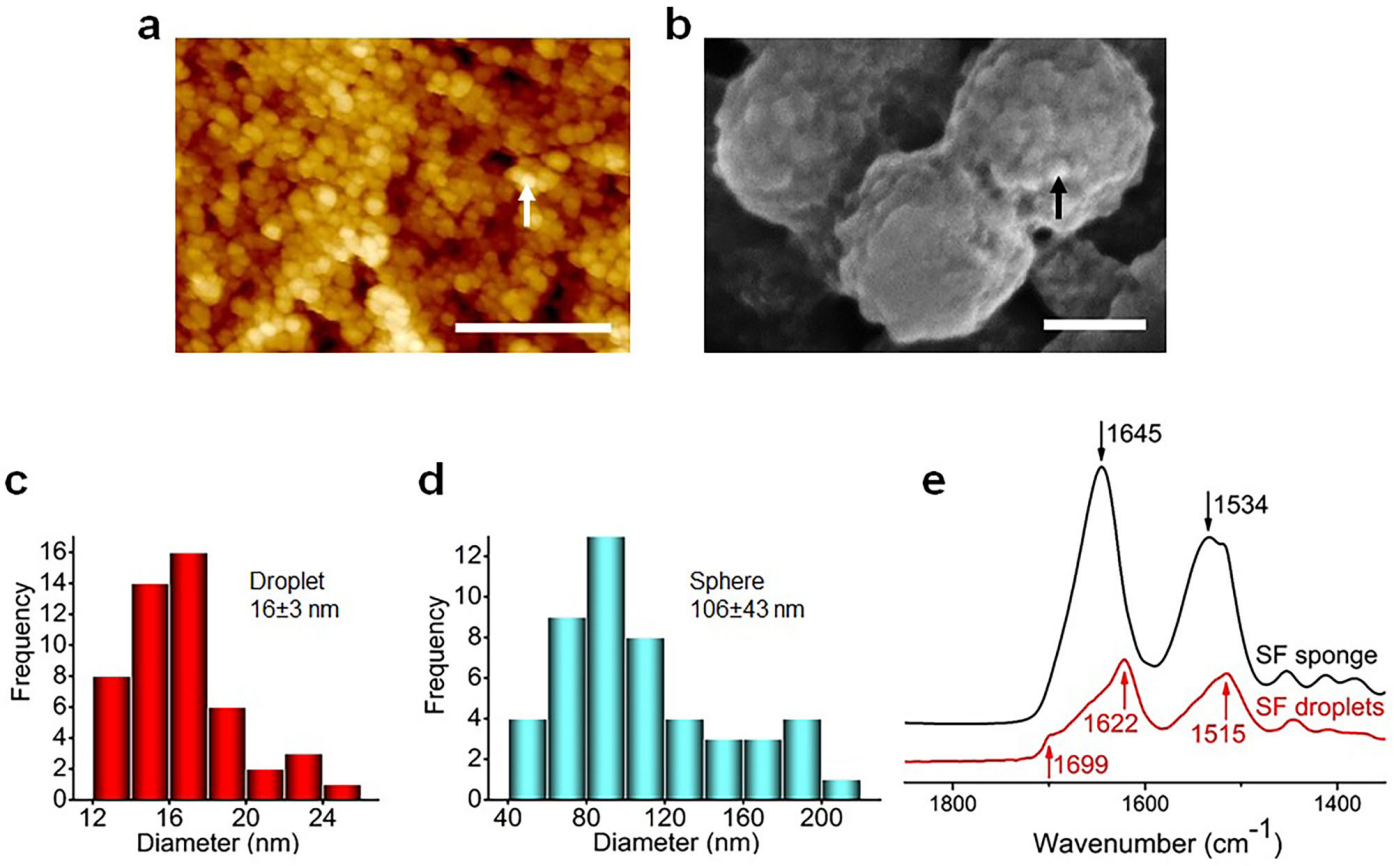
Morphology, size, and structure of crystallized silk fibroin (SF) droplets/spheres. **a** AFM and **b** SEM images at different magnifications showing the morphology of SF spheres consisting of smaller SF droplets. The white arrow in **a** and the black arrow in **b** respectively indicate the morphology of one single SF sphere and one single SF droplet in one sphere. Scale bars: 2 μm and 100 nm in **a** and **b**, respectively. **c** and **d** are the size distribution of droplets and spheres, respectively. **e** ATR-FTIR spectra revealing the dominant random-coil structure of SF sponges obtained by freeze-drying the SF aqueous solution (used to produce the SF droplets) and the β-sheet-dominant crystalline structure of SF droplets collected from the ethanol bath.

When aqueous SF solution droplets were electrosprayed into an ethanol bath, crystallized SF droplets formed, which further self-aggregated to form larger spheres (Fig. 1b). The morphology of SF spheres made up of SF droplets is shown in Fig. 2a,b and Supplementary Fig. 1. The SEM images clearly demonstrate that each sphere is an assembly of smaller droplets (Fig. 2b and Supplementary Fig. 1b). The average diameter of the SF droplets and spheres are 16 ± 3 and 106 ± 43 nm, respectively (Fig. 2c,d). The secondary structure of the SF droplets/spheres was examined using ATR-FTIR. For comparison, SF sponge obtained by freeze-drying the aqueous SF solution (used to produce the SF droplets) was adopted as a control. Figure 2e shows that the SF sponge has characteristic absorption peaks at around 1645 and 1534 cm^−1^, suggesting the dominant random-coil structure of SF^16–18^. However, the SF droplets/spheres collected from the ethanol bath present characteristic absorption peaks at around 1699, 1622, and 1515 cm^−1^, indicating the β-sheet-dominant crystalline structure of SF^16–18^. The findings indicate that the crystallization of SF droplets happened in the ethanol bath, not in the aqueous solution.

### Hierarchical SF fibers self-assembled from crystallized SF droplets

**Fig. 3:**
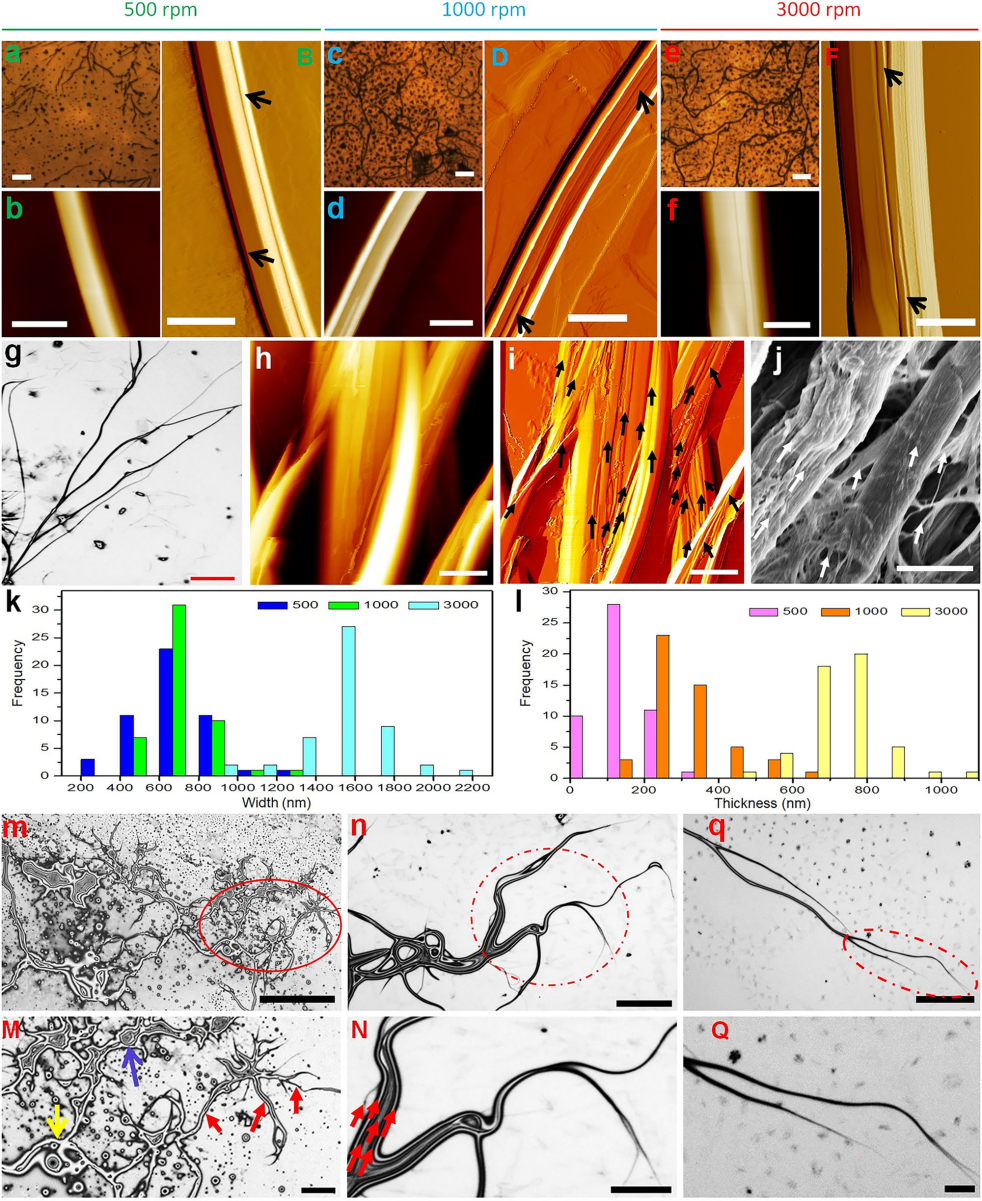
Morphology, structure, size, and assembly mechanism of hierarchical silk fibroin (SF) fibers self-assembled from the crystallized SF droplets in the rotary assembly system. **a**, **c** and **e** Microscopic images showing the morphology of hierarchical SF fibers assembled at different rotating speeds for 5 min, respectively. (**b**) (**B**), (**d**) (**D**), and (**f**) (**F**) AFM images showing the detailed morphology and structure of the hierarchical SF fibers obtained at different rotating speeds for 5 min, respectively (**b**, **d**, and **f** from the Height Channel of AFM; **B**, **D**, and **F** from the Peak Force Error Channel of AFM). The arrows in **B**, **D**, and **F** indicate the fibrils in the hierarchical SF fibers. **g** Microscopic morphology of hierarchical SF fibers in a branched network assembled at 3000 rpm for 5 min. **h**, **i** The profiles of fibrils in the hierarchical SF fibers assembled at 3000 rpm for 5 min after the fibrils were released by ultrasonic treatment (**h** from the Height Channel of AFM and **i** from the Peak Force Error Channel of AFM; the black arrows indicating the fibrils aligned in the long axis of the fibers). **j** SEM morphology of natural SF fibers with fibrils released by ultrasonic treatment. The white arrows indicate the fibrils aligned in the long axis of the fibers. **k** Width and **l** thickness distribution of hierarchical fibers assembled at different rotating speeds (500, 1000, and 3000 rpm) for 5 min, respectively. **m**, **n**, **q** Microscopic images of the resultant assemblies after the crystallized SF droplets being induced in the rotary assembly system at a rotating speed of 3000 rpm for 1, 2, and 3 min, respectively. **M**, **N**, **Q** are the magnifications of the circled parts in **m**, **n**, **q**, respectively. The yellow, blue, and red arrows in **M** indicate the coalescing SF droplets, the assembly formed by the coalesced SF droplets and the fibers sprouting from the assembly of coalesced SF droplets, respectively. The red arrows in **N** indicating the aligned structures in the resultant assembly. Scale bars: 10 μm in **a**, **c**, **g**, **j**; 20 μm in **e**; 1000 nm in **b**, **B**, **d**, **D**, **f**, **F**, **h** and **i**; 250, 25, and 25 μm in **m**, **n**, and **q**, respectively; 50, 10, and 5 μm in **M**, **N**, and **Q**, respectively.

To investigate the feasibility of the crystallized SF droplets to self-assemble into hierarchical suprafibrillar silk fibers, the mixtures of SF droplets/spheres and ethanol were induced using a circular rotary duct (with an inner radius ~1.5 mm), operated at 500, 1000, and 3000 rpm for 5 min, respectively (the rotator has a radius ~6 cm; Fig. 1c). At a low rotating speed of 500 rpm, some short and branched fibers were observed (Fig. 3a and Supplementary Fig. 2a). The as-assembled SF fibers have an average width of 700 ± 172 nm, with an average thickness of 158 ± 60 nm (Fig. 3k,l). The fibrils in the fiber are indicated by the black arrows in Fig. 3B. With an increase in the rotating speed to 1000 rpm, many long and branched fibers were observed (Fig. 3c and Supplementary Fig. 2b). Most of the fibers have a width between 600 and 800 nm, with a thickness between 200 and 400 nm (Fig. 3k,l). The average width and thickness of the fibers increased to 746 ± 137 nm and 319 ± 89 nm, respectively. Interestingly, nanofibrils aligned in the long-axis direction of the fibers were clearly observed, as indicated by the black arrows in Fig. 3D. With the rotating speed of 3000 rpm, more and longer branched fibers were produced (Fig. 3e and Supplementary Fig. 2c). The clearer branched profile of fibers can be seen in Fig. 3g. Obviously, the crystallized SF droplets self-assembled into a hierarchical network of branched fibers, similar to that of capillaries in our body (Fig. 3g). The width of most of fibers is in the range of 1400–1600 nm (average width 1496 ± 216 nm), with a thickness between 600 and 800 nm (average thickness 710 ± 95 nm; Fig. 3k,l). This could be due to the assembly of more droplets onto the existing fibers to induce fiber growth in both the radial and longitudinal directions with an increase in rotating speed. Clearly, in the resultant fibers, nanofibrils (width ~65 nm) aligned along the long axis of fibers were observed, as shown by the black arrows in Fig. 3F. Clearer profiles of fibrils in the fibers can be found in Fig. 3h,i (the fibrils are clearly indicated by the black arrows in Fig. 3i). The hierarchical suprafibrillar structure of the as-assembled fibers is very similar to that of natural silk fibers (Fig. 3j). The self-assembly process can also be programmed by adjusting the rotary inducing-treatment duration to fabricate hierarchical suprafibrillar SF fibers with varied width and thickness (Supplementary Fig. 3).

To gain insight into the assembly mechanism, the self-assembly process was monitored by investigating the resultant structures at the rotating speed of 3000 rpm after 1, 2, and 3 min, respectively. As shown in Fig. 3m,M, the crystallized SF droplets/spheres coalesced into larger assemblies after 1 min of the rotary inducing-treatment (The yellow, blue, and red arrows in Fig. 3M indicate the coalescing SF droplets, the assembly from the coalesced SF droplets and the fibers sprouting from the assembly of coalesced SF droplets, respectively.). This is similar to the coalescence of liquid crystalline droplets of spidroin prior to the formation of the silk fiber in the gland of spiders (Fig. 1a)^11^. In the spider’s silk spinning, the coalesced assemblies were subsequently aligned and elongated along the narrowing spinneret to form one single suprafibrillar silk fiber under dominant shearing force (Fig. 1a)^11^. Different from the spider’s spinning, here, the assemblies of coalesced SF droplets started to sprout to form fibers under dominant centrifugal force and shearing force, as indicated by the red arrows in Fig. 3M after 1 min of the rotary inducing-treatment. When the treatment duration was increased to 2 min, the assemblies further sprouted to form networks of branched suprafibrillar fibers, which is similar to the formation of capillaries in our body (Fig. 3n,N). In this assembly system, ethanol cannot dissolve SF, just inducing its structural transformation from dominant random coils to β-sheet-dominant crystalline structure (Fig. 2e)^16^. Therefore, the crystallized SF droplets/spheres and ethanol were two different phases before and after the rotary inducing-treatment, which is very important for promoting the coalescence of SF droplets under the centrifugal force and shearing force (Fig. 3m,M). When the centrifugal force and shearing force exceeded the surface tension of the SF phase and the friction force from the ethanol phase, the resultant assemblies of coalesced SF droplets started to sprout to form fibers. During the sprouting process, the assembly was significantly aligned (in which the aligned structures were clearly observed, as indicated by the red arrows in Fig. 3N). Under the centrifugal force and shearing force, the formed fibers further sprouted, and elongated into smaller fibers after 3 min (Fig. 3q,Q). The findings indicate that the formation of hierarchical suprafibrillar silk fibers in the rotary assembly system is a process containing the sprouting, alignment, and elongation of coalesced SF droplets. Here, we also used crystallized solid SF particles instead of the crystallized SF droplets for the same treatment, but failed to get the hierarchical suprafibrillar structure (Supplementary Fig. 4). It is hence reasonable to conclude that including SF molecules in crystallized soft droplets can help the formation of hierarchical suprafibrillar SF fibers. This assembly system allows us to fabricate hierarchical suprafibrillar silk fibers with width and thickness varying from micrometers down to even nanometers by increasing the inducing-treatment duration (Supplementary Fig. 3) or decreasing the rotating speed (Fig. 3k,l), which is very hard to be achieved by the spinning technology, especially at a such low concentration of SF solution (0.2%, wt/v). With the small size, the as-assembled branched hierarchical suprafibrillar silk fibers would find lots of applications in different fields^19–21^. Our work provides a facile strategy to fabricate hierarchical suprafibrillar silk fibers with small sizes using very low concentrations of SF solution and could help to understand the natural silk spinning mechanism of spiders through lateral thinking.

### Mechanical properties of self-assembled hierarchical SF fibers

**Fig. 4:**
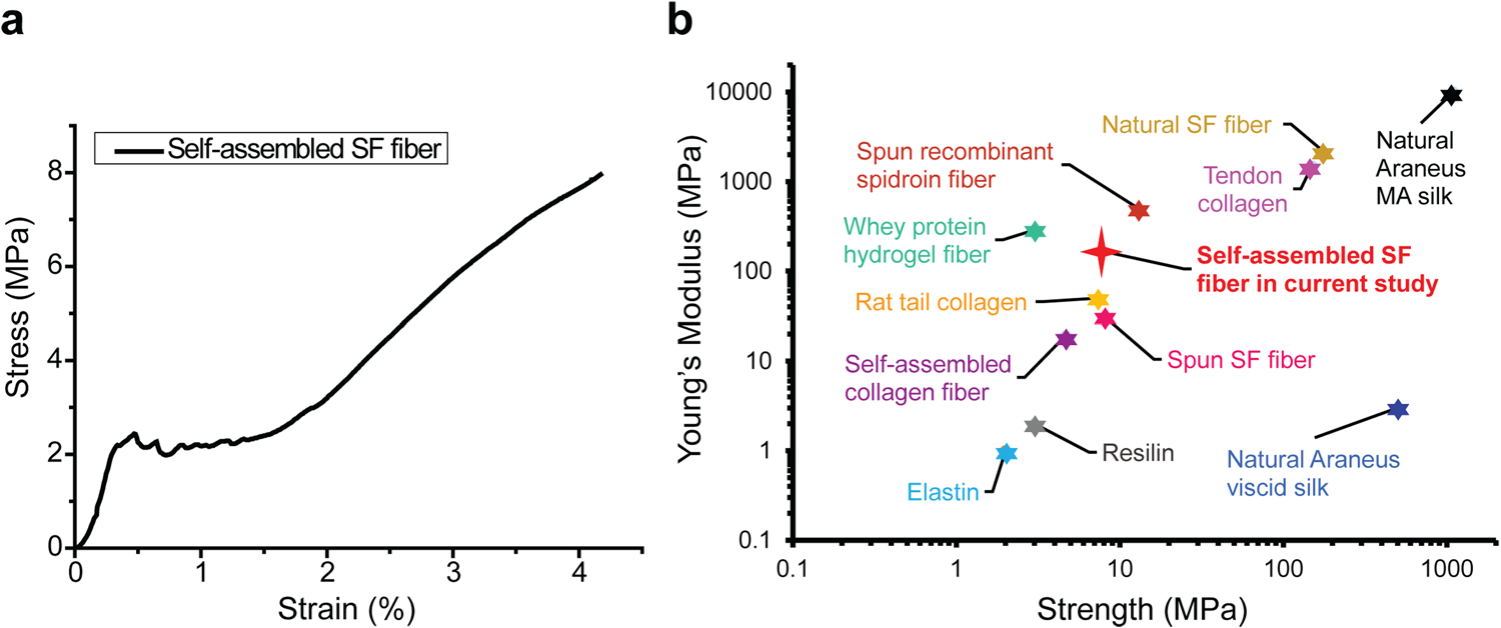
Mechanical characteristics of self-assembled hierarchical silk fibroin (SF) fibers. **a** Representative stress-strain curve of the hierarchical SF fibers assembled at 3000 rpm for 5 min. **b** Comparison of Young’s modulus and strength of the hierarchical SF fibers (assembled at 3000 rpm for 5 min) with some representative protein fibers and materials (the data on the mechanical characteristics of natural SF fibers were obtained in this work)^12,22–24,28^.

To investigate the mechanical properties of the as-assembled hierarchical SF fibers, the representative stress–strain curve of the fibers (assembled at 3000 rpm for 5 min) is presented in Fig. 4a. The self-assembled SF fibers displayed mechanical characteristics of soft materials with the tensile strength, strain, and Young’s modulus ~8 MPa, ~4% and ~166 MPa, respectively. Compared to the natural SF fibers (Supplementary Fig. 5; the fabrication method in “Generation of SF solution” in the Methods section), the self-assembled SF fibers (at 3000 rpm for 5 min) in this work has a lower Young’s modulus (Fig. 4b), which could be attributed to different concentrations of SF solution used for production of these two classes of fibers, respectively. In this work, the concentration of SF solution used to assemble the hierarchical SF fibers is just 0.2% (wt/v). However, in the natural silk spinning, the concentration of SF solution is up to ~30% (wt/v)^10^, which is ~150 times higher than that of the SF solution used in this work. Noteworthily, the self-assembled SF fibers show a greater Young’s modulus than some other protein fibers or materials (such as the self-assembled collagen fibers, rat tail collagen, elastin, and resilin as well as the natural Araneus viscid silks)^22,23^. In particular, the Young’s modulus (166 MPa) of the as-assembled SF fibers is significantly greater than that (31.2 MPa) of artificially spun SF fibers from a high concentration (18%, w/w) of SF solution, with an average fiber width (1940 ± 690 nm) close to that of the as-assembled SF fibers^24^. Currently, the fabrication of artificial SF fibers mainly relies on the spinning technology. However, the spinning usually needs a concentration of SF solution around 15% (w/w) or more^10,24^, which is significantly higher than that (0.2%, wt/v) of SF solution used in this work. Furthermore, it is hard to reduce the width or thickness of SF fibers down to the sub-micrometer and nanometer scale by spinning. The self-assembly technology developed in this work could be a good alternative for artificial fabrication of hierarchical suprafibrillar SF fibers with small width and thickness at a very low concentration of SF solution. Although the self-assembled SF fibers in our work are not very strong in mechanical properties, the tensile strength (8 MPa) is comparable to that of some human soft tissues such as skin and cartilage (4–5 MPa and 3.7–10.5 MPa for the tensile strength range of human skin and cartilage, respectively)^25,26^. Furthermore, the Young’s modulus (166 MPa) is significantly higher than that of human skin and cartilage (5 kPa–140 MPa and 0.7–15.3 MPa for the Young’s modulus range of human skin and cartilage, respectively)^26,27^. Therefore, as a kind of soft biomaterials, the mechanical properties of self-assembled SF fibers in our work are good for use in cell culture and regeneration of soft tissues such as human skin and cartilage.

### Bioactivity of self-assembled hierarchical SF fibers

**Fig. 5:**
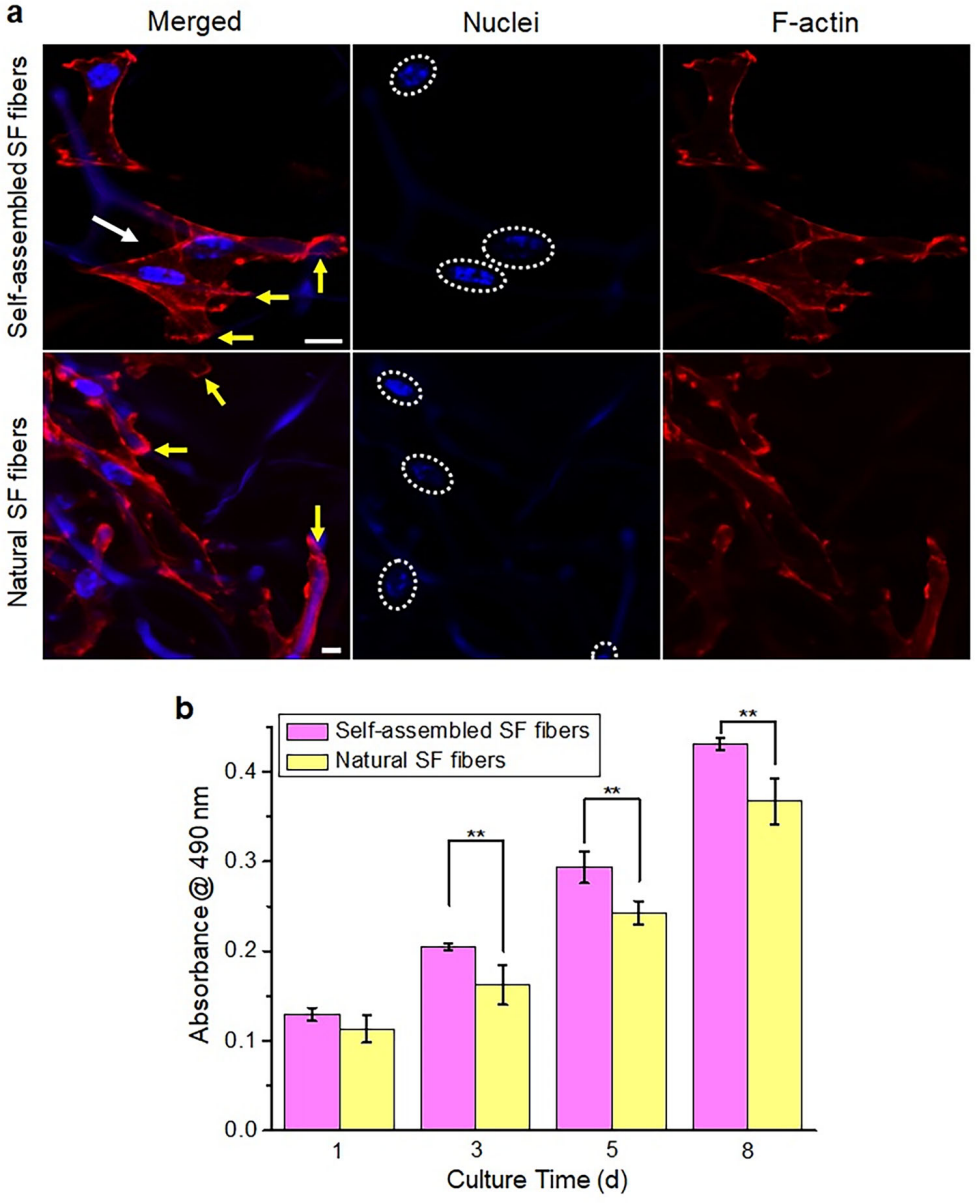
Bioactivity of self-assembled hierarchical silk fibroin (SF) fibers. **a** Growth of human umbilical vein endothelial cells (HUVECs) on the self-assembled (at 3000 rpm for 5 min) and natural SF fibers, respectively (cell nuclei and fibers were stained in blue where the nuclei with bright-blue dots are indicated by the white dotted ovals. F-actin was stained in red. The white arrow indicates the interaction between cells on different fibers. The yellow arrows indicate the different cell-fiber interactions on the self-assembled and natural SF fibers, respectively.). Scale bars: 10 μm. **b** Viability (the cell viability is proportional to the absorbance level) of HUVECs on the self-assembled (at 3000 rpm for 5 min) and natural SF fibers, respectively, at fixed culture time points. Error bars represent standard deviation (*n* = 4). (^**^p < 0.01).

With the hierarchical suprafibrillar structure similar to that of collagen fibers of natural extracellular matrix and good biocompatibility of SF^16,29–33^, the self-assembled SF fibers are expected to have applications in the biomedical field. In this study, human umbilical vein endothelial cells (HUVECs) were used to investigate the bioactivity of the hierarchical SF fibers (assembled at 3000 rpm for 5 min) with the natural SF fibers as a control (Supplementary Fig. 5; the fabrication method in “Generation of SF solution” in the Methods section). An equal quantity of HUVEC cells were seeded and cultured on the surface of coverslips deposited with one thin layer of either the natural SF fibers or the self-assembled SF fibers (please see the Methods section for more details). After 4 days of culture, the HUVEC cells were fixed and stained to image their actin stress fibers and nuclei for observing the cytoskeletal organization. As shown by the confocal microscopy images in Fig. 5a, the HUVEC cells demonstrated well-developed actin cytoskeletal organization on the self-assembled SF fibers with an extensively spreading cell morphology. Cells had spanned single fibers to interact with the cells on the adjacent fibers, as shown by the white arrow on the merged image of the self-assembled SF fibers group in Fig. 5a. In contrast, the actin cytoskeletal profile became less-pronounced in the HUVEC cells growing on the natural SF fibers (Fig. 5a). Obviously, the cells merely grew on local single fibers with a less-spread morphology and blunt cell edges, as indicated by the yellow arrows on the merged image of the natural SF fibers group (Fig. 5a). In this work, the crystallized SF droplets self-assembled into branched networks of hierarchical suprafibrillar SF fibers, not single SF fibers (Fig. 3e–i). This kind of self-assembled SF fibrous networks played an important role in stabilizing and supporting cells for extensive spreading and fibers-spanned growth where the fibers still remained good morphology (Fig. 5a; The yellow arrows indicate the interaction of cells and fibers, and the white arrow indicates the interaction between cells on different fibers on the merged image of the self-assembled SF fibers group.). The growth viability of cells was further quantified by the classic MTS assay in which the MTS absorbance level is proportional to the cell viability (Fig. 5b). With an increase in culture time, the cell viability increased on both the natural and self-assembled SF fibers, indicating that both types of fibrous substrates can promote the growth of cells. Noticeably, the cells on the self-assembled SF fibers displayed significantly higher viability compared to those on the natural SF fibers, especially with longer culture periods (3, 5, and 8 days; Fig. 5b). This could be due to that the fibrous network of branched suprafibrillar SF fibers, with a similar structure to that of the natural extracellular matrix, provides a better micro-environment for growth of cells^19,31^. These encouraging findings would facilitate the design and development of hierarchical fibers-based biomaterials for applications in biomedicine.

In summary, hierarchical suprafibrillar silk fibers were successfully developed by the bioinspired self-assembly of crystallized aqueous SF droplets. The findings reveal that the formation of branched hierarchical silk fibers induced by the rotary inducing-treatment is a process of coalescent SF droplets sprouting to form branched fibrous networks, different from the liquid crystalline spinning of spiders in which one single silk fiber is obtained. The self-assembly process can be programmed by adjusting the assembly parameters to produce branched hierarchical suprafibrillar silk fibers at the micrometer and even nanometer scale. The as-assembled silk fibers show mechanical characteristics of soft biomaterials and can significantly promote the spreading and growth of HUVECs compared to the natural SF fibers. With the good bioactivity, small width and thickness, and hierarchical suprafibrillar structure similar to that of collagen fibers in the natural extracellular matrix, the self-assembled branched silk fibers could find various applications in different fields, especially in biomedicine. This work provides a facile strategy for developing hierarchical silk fibers for various applications and may help to understand the liquid crystalline spinning of spiders.

## Methods

### Generation of SF solution

The outer coating sericin of natural silk fibers was removed by boiling (four times, 20 min/time) silk cocoons in 0.5% (w/v) Na_2_CO_3_ aqueous solution. Then the silk fibers were washed in ultrapure water to remove the residual sericin. After drying, the sericin-free SF fibers were obtained and then dissolved in a mixture of CaCl_2_, H_2_O, and CH_3_CH_2_OH (molar ratio: 1:8:2) at 65 °C. The as-prepared solution was dialyzed in ultrapure water with cellulose tubes (molecular weight cut-off: 14 kDa; Sigma Aldrich, Australia) at room temperature for 4 days. Following filtering and centrifuging, SF sponge was obtained by freeze-drying the resultant solution with a freeze dryer (FreeZone 2.5 Liter Benchtop Freeze Dryer; Labconco, Kansas City, MO, USA). The SF solution (0.2%) was produced by dissolving 0.2 g of SF sponge in 100 mL of ultrapure water.

### Preparation of crystallized SF droplets

The crystallized SF droplets were produced by electrospraying 0.5 mL of the aqueous SF solution into an ethanol bath to obtain 30 mL of mixture. A plastic syringe with a blunt metal needle (gauge 26) was used. The flow rate (0.1 mL h^−1^) was controlled by a syringe pump (KD Scientific), and a voltage of 16 kV was applied to the metal syringe needle by a high-voltage power supply (Gamma High Voltage). The ethanol bath was placed 20 cm away from the needle tip (Fig. 1b).

### Assembly of hierarchical suprafibrillar silk fibers

1 mL of the SF droplets/ethanol mixture obtained from the electrospraying was put in each of circular rotary ducts (~1.5-mm inner radius). The solutions were treated in the rotary assembly system at 500, 1000, and 3000 rpm for 5 min, respectively, and 3000 rpm for 1, 2, 3, and 10 min, respectively, to induce the self-assembly of the SF droplets at ambient temperature using a self-made rotator (~6 cm radius). After treatments, the resultant structures were used for further characterizations.

### Characterizations

Fourier transform infrared (FTIR) spectra of the SF sponge fabricated by freeze-drying the aqueous SF solution (used to produce the SF droplets) and the crystallized SF droplets/spheres were obtained on a Bruker VERTEX 70v instrument using the attenuated total reflectance (ATR) mode (4 cm^−1^ resolution, 64 scans). The morphology of crystallized SF droplets/spheres and SF fibers was shown using a Bruker MultiMode 8 atomic force microscope (AFM) in the ScanAsyst-Air imaging mode with Silicon Nitride probes (Bruker) and/or a scanning electron microscope (SEM) (Zeiss Supra 55 VP). The width and thickness of fibers (*n* = 50) as well as the width of fibrils (*n* = 50) were analyzed using the NanoScope Analysis software (Bruker). The diameter of crystallized SF droplets/spheres (*n* = 50) was calculated from representative SEM images using an image processing software (ImageJ). The morphology of fibers was also characterized with a DP71 microscope (Olympus). For the mechanical test, single natural SF fibers (*n* = 10) and the assembled SF fibers (*n* = 10) were carefully mounted on testing templates, respectively, which were then carefully mounted into the Agilent T150 UTM facility (Agilent Technologies Inc., USA). The test was conducted at a strain rate of 1 × 10^−3^ s^−1^ until the fiber fractured in a standard condition lab (20 ± 2 °C and 65 ± 2% relative humidity). Tensile stress and strain were calculated and plotted, and the Young’s modulus was calculated as the slope of initial linear section of the stress–strain curve.

### Cell culture and seeding

The HUVEC cells (Life Technologies, Australia) were cultured in the Medium 200 added with the low serum growth supplement (Life Technologies, Australia). The natural and self-assembled suprafibrillar SF fibers were coated onto coverslips, respectively, to form one thin layer of substrate. After sterilization, the coated coverslips were placed in 24-well plates (Greiner Bio-One) for cell culture. Then the HUVEC cells were evenly seeded onto the coated coverslips at a density of 2.3 × 10^4^/well and maintained in vitro under standard culture conditions (37 °C, 5% CO_2_).

### Assay of cell growth and viability

At fixed culture time points (1, 3, 5, and 8 days) after cell seeding, the viability of HUVEC cells on the fibrous substrates was determined using MTS assay (Promega, USA) according to the manufacturer’s instruction with absorbance measured at 490 nm using a microplate reader (SH-1000Lab, Corona Electric Co., Ltd, Japan). Following 4 days of culture, the composites of cells/coated coverslips were rinsed using PBS and then fixed in 4% paraformaldehyde (Sigma-Aldrich, Australia) for 30 min at room temperature, followed by rinsing with PBS. Subsequently, the composites were permeabilized using 0.1% Triton X-100 (Sigma-Aldrich, Australia) for 10 min. After rinsing with PBS, the composites were incubated with Image-iT^®^ FX Signal Enhancer Ready Probes^™^ reagent (Life Technologies, Australia) for 30 min, followed by being rinsed with PBS, and then were incubated in Alexa Fluor^®^ 568 Phalloidin (1:100; Life Technologies, Australia) in dark for 1 h. Following being rinsed with PBS, the composites were incubated with DAPI (Life Technologies, Australia) in dark for 10 min. The resultant samples were assessed with a confocal fluorescence microscope (Leica TCS SP5 Confocal Microscope, Leica Microsystems, Wetzlar).

### Statistical analysis

All experiments were performed in at least triplicates. Data were expressed as mean ± standard deviation. Statistical difference was determined using one-way ANOVA in an Origin 9 software (OriginLab, USA). Difference with *p* < 0.01 was considered as statistical significance.

## Supplementary information

Supplementary Information

## Data Availability

The data supporting this study are in the paper and the Supplementary Information or available from the corresponding authors on reasonable request.
