# Supplementary material for: Bioactive hierarchical silk fibers created by bioinspired self-assembly
Source: Nat Commun. 2021 Apr 22;12:2375. doi: 10.1038/s41467-021-22673-4 (PMC8062673; doi:10.1038/s41467-021-22673-4)
Supplement: Supplementary file 1 — Supplementary Information [file 41467_2021_22673_MOESM1_ESM.pdf]

Supplementary Information

Bioactive Hierarchical Silk Fibers Created by Bioinspired Self-Assembly

Linpeng Fan, Jing-Liang Li, Zengxiao Cai & Xungai Wang

Institute for Frontier Materials, Deakin University, Geelong, VIC 3216, Australia

Supplementary Figures

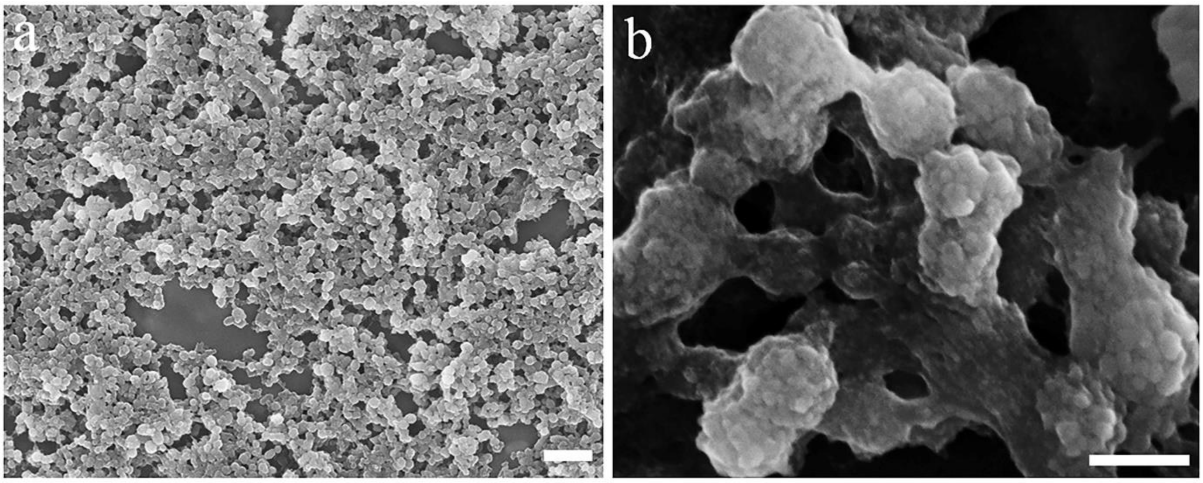

Supplementary Fig. 1 Morphology of crystallized silk fibroin (SF) droplets/spheres fabricated in this work. (a) An SEM image of SF spheres made of smaller droplets at a low magnification. (b) An SEM image of SF spheres made of smaller droplets at a high magnification. Scale bars: 1000 and 200 nm in (a) and (b), respectively.

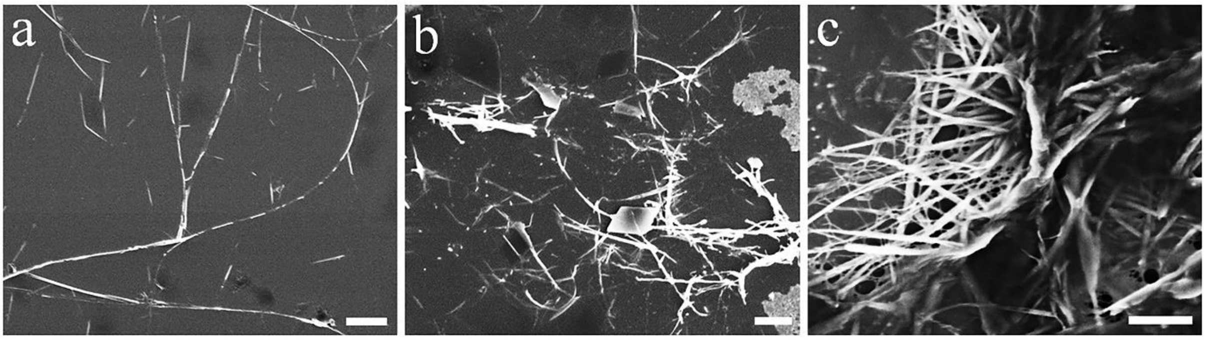

Supplementary Fig. 2 SEM morphology of the self-assembled silk fibroin fibers after rotary inducing-treatment for 5 minutes at different rotating speeds of (a) 500 rpm, (b) 1000 rpm and (c) 3000 rpm. Scale bars: 20, 10 and 15 μm in (a), (b) and (c), respectively.

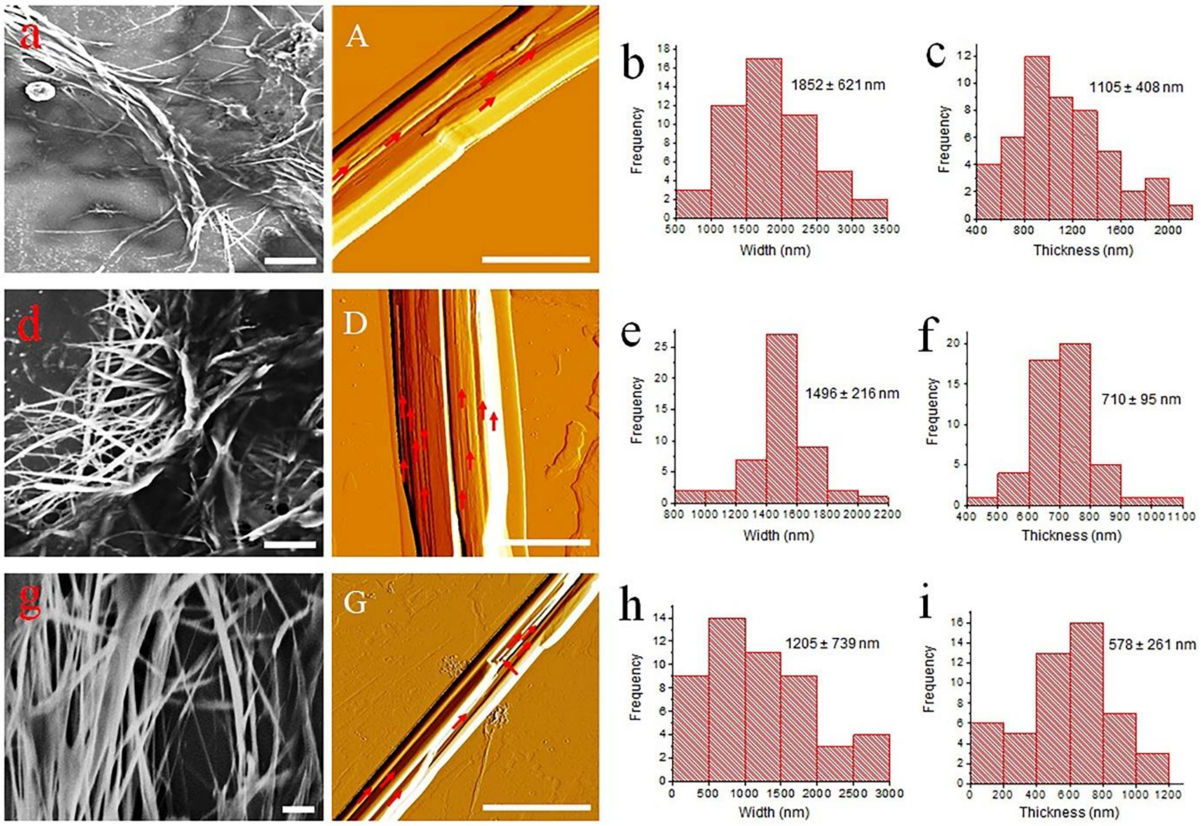

Supplementary Fig. 3 Morphology, and width and thickness distribution of the self-assembled silk fibroin fibers after rotary inducing-treatment at a rotating speed of 3000 rpm for (a-c) 3 minutes, (d-f) 5 minutes and (g-i) 10 minutes. Scale bars: 20, 4, 15, 2, 10, and 4 μm in (a), (A), (d), (D), (g) and (G), respectively.

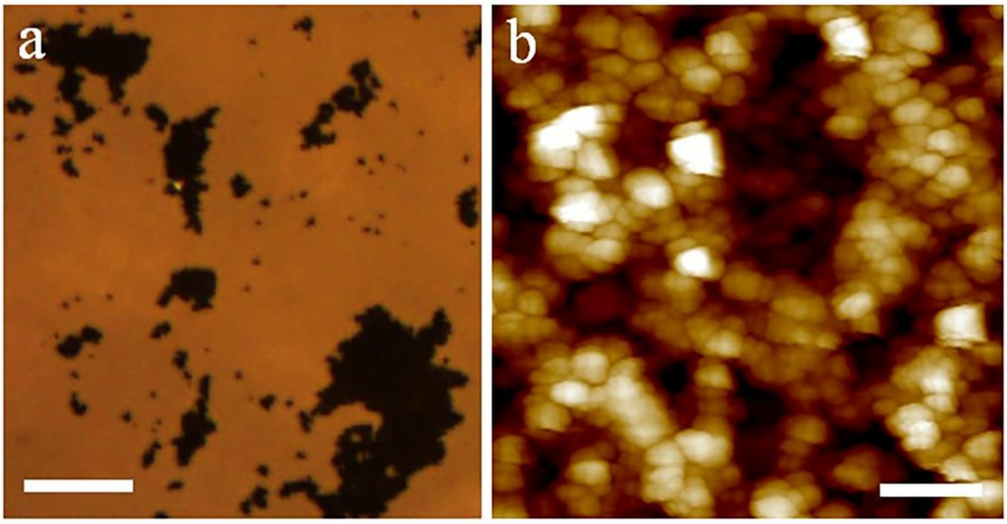

Supplementary Fig. 4 The resultant structures after the mixture of solid silk fibroin particles/ethanol being treated using the rotary assembly system (developed in this work) with a speed of 3000 rpm for 10 minutes. (a) A microscopic image. (b) An AFM image. Scale bars: 65 μm and 1000 nm in (a) and (b), respectively.

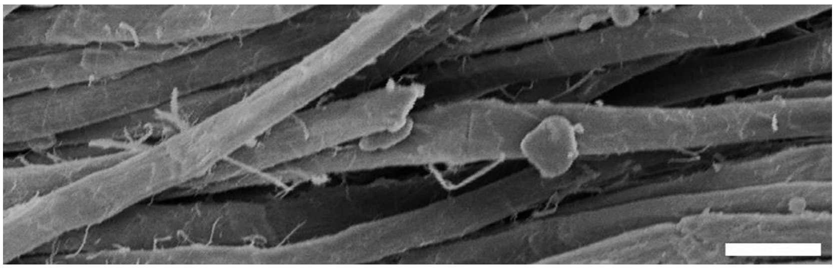

Supplementary Fig. 5 SEM morphology of natural silk fibroin fibers. Scale bar: 20 μm.
